# Identification of Human B-1 Helper T Cells With a Th1-Like Memory Phenotype and High Integrin CD49d Expression

**DOI:** 10.3389/fimmu.2018.01617

**Published:** 2018-07-16

**Authors:** Jae-Ghi Lee, Joon Young Jang, Taishi Fang, Yixuan Xu, Ji-Jing Yan, Jung-Hwa Ryu, Hee Jung Jeon, Tai Yeon Koo, Dong Ki Kim, Kook-Hwan Oh, Tae Jin Kim, Jaeseok Yang

**Affiliations:** ^1^Transplantation Research Institute, Seoul National University College of Medicine, Seoul, South Korea; ^2^Transplantation Center, Seoul National University Hospital, Seoul, South Korea; ^3^Department of Internal Medicine, Hallym University College of Medicine, Seoul, South Korea; ^4^Department of Internal Medicine, Seoul National University College of Medicine, Seoul, South Korea; ^5^Division of Immunobiology, Sungkyunkwan University School of Medicine, Suwon, South Korea; ^6^Department of Surgery, Seoul National University Hospital, Seoul, South Korea

**Keywords:** antibody, B-1 cell, CD49d, helper T cell, memory phenotype, Th1

## Abstract

Human B-1 cells have been proposed to be CD20^+^CD27^+^CD43^+^CD1c^−^ B cells found in the umbilical cord and adult peripheral blood, but their regulatory mechanisms have not been well elucidated. Previously, we reported that mouse CD49d^high^ CD4^+^ T cells could enhance the secretion of natural antibodies by B-1 cells. In this study, we aimed to investigate the presence and helper functions of the human equivalents of murine CD49d^high^ CD4^+^ T cells. Here, we showed that human CD49d^high^ CD4^+^ T cells found in the peritoneal cavity (PEC), spleen, and peripheral blood can enhance the production of IgM antibodies by B-1 cells. As revealed in mouse, CD49d^high^ CD4^+^ T cells were more abundant in the PEC and showed a higher tendency to form conjugates with B cells than CD49d^low^ CD4^+^ T cells. Moreover, CD49d^high^ CD4^+^ T cells showed a Th1-like memory phenotype, characterized by high expression of CD44 and CXCR3; low expression of CD62L and CCR7; rapid production of IFN-γ, tumor necrosis factor-α, and IL-2 upon stimulation with phorbol myristate acetate and ionomycin; and rapid proliferation upon stimulation with anti-CD3 and anti-CD28 antibodies. These cells also expressed high levels of PD-1, ICOS, and CD5, suggesting that they are undergoing chronic stimulation. Remarkably, CD49d^high^ CD4^+^ T cells specifically helped B-1 cells, but not follicular memory B cells (CD27^+^ CD43^−^CD1c^−^) or marginal zone B cells (CD27^+^CD43^−^CD1c^+^), produce IgM and IgG antibodies. In parallel, the titer of human anti-blood group A IgM was positively correlated with the frequency of CD49d^high^ CD4^+^ T cells. In conclusion, we identified human CD49d^high^ CD4^+^ T cells with a Th1-like memory phenotype that secrete Th1 proinflammatory cytokines and help B-1 cells secrete antibodies, thereby aiding in primary defense. We suggest that these CD49d^high^ CD4^+^ T cells are a unique type of B-cell helper T cells distinct from follicular helper T cells.

## Introduction

B-1 cells comprise a fetal progenitor-derived self-renewing population of B cells that generate natural antibodies as a primary defense against microbial infection (1, 2). Although B-1 cells have been well investigated in mice, human B-1 cells have not been well studied; however, these cells have recently been identified as CD3^−^CD19^+^CD20^+^CD27^+^CD43^+^CD69^−^CD70^−^ cells based on their fundamental B-1 cell functions, such as spontaneous IgM secretion, efficient T cell stimulation, and tonic B cell receptor-mediated signaling (3). The majority of mouse B-1 cells resides in the serosal cavities, and a small proportion of B-1 cells recirculate through the blood, spleen (SP), and other lymphoid tissues and enter into peripheral inflammatory sites. B-1 cells are known to function in a T cell-independent manner and are usually activated through innate immunity receptors such as TLR4 and TLR9 (4, 5), whereas conventional B-2 cells require help from CD4^+^ T cells for differentiation into memory B or plasma cells and produce high-affinity antibodies. However, mouse B-1 cells are excellent antigen-presenting cells for CD4^+^ T cells (6), and the ability of human B-1 cells to support the proliferation of T cells has also been confirmed (7). Moreover, the active interaction between B-1 and CD4^+^ T cells may activate reverse signaling for B-1 cell help from CD4^+^ T cells, as is the case in mouse B-1a cells (8, 9). Therefore, it would be interesting to determine whether human CD4^+^ T cells are able to provide help for human B-1 cells, as well as which kinds of human CD4^+^ T cells are responsible for this B-1 helper function.

Previously, our group reported that mouse peritoneal CD49d^high^ CD4^+^ T cells were able to help B-1a cells produce natural antibodies (8). These cells showed a memory phenotype similar to CD44^high^ CD62L^low^ cells and rapidly released Th1 cytokines such as IFN-γ and tumor necrosis factor (TNF)-α in response to stimulation with phorbol myristate acetate (PMA) and ionomycin or anti-CD3 and anti-CD28 (8). They developed very early (within 2 weeks of age), showed stem-cell like properties, and expressed high levels of PD-1, ICOS, and CXCR3 (8). However, it is not yet known whether there are human equivalents of these CD4^+^ T cells.

Memory T cells are generated through previous events of antigen-specific T cell activation, proliferation, and differentiation, and they induce stronger and more rapid responses in secondary immune reactions than naive T cells (10, 11). As some T cells such as NKT cells obtain a memory phenotype during the course of development, these T cells are referred to as innate T cells, since they are not generated through peripheral expansion upon antigenic encounters (12). In addition to NKT cells, there are other types of innate T cells, such as a certain type of T cells that develop by interactions of the self-antigen/MHC complex with low-affinity T cell receptors, mainly under lymphopenic conditions (13, 14). These developmental processes require IL-7 and IL-15, which induce T-bet and Eomes, respectively (15, 16). We hypothesize that the mouse peritoneal CD49d^high^ CD4^+^ T cells are another type of innate CD4^+^ T cell, since they develop very early, even after neonatal thymectomy at day 3 (8).

In this study, we aimed to investigate the presence and functions of the human equivalents of murine CD49d^high^ CD4^+^ T cells. We found that CD49d^high^ CD4^+^ T cells were present in the human peritoneal cavity (PEC), SP, and peripheral blood (PB), and we also revealed that they exhibited a Th1-like memory phenotype, similar to those of mouse CD49d^high^ CD4^+^ T cells. Notably, these human CD49d^high^ CD4^+^ T cells were found to help recently identified human B-1 cells, but not other types of human B cells, secrete antibodies.

## Materials and Methods

### Sample Preparation

The human peritoneal fluid, SP, and PB samples were collected from patients who initiated peritoneal dialysis, organ donors with brain-death, and healthy volunteers, respectively. Peritoneal cells were obtained by centrifugation of the whole peritoneal fluid. Splenocytes were obtained through mechanical disruption and passage of the homogenate though a nylon membrane. PB mononuclear cells were obtained by density gradient separation using Ficoll (GE Healthcare, Cleveland, OH, USA).

### Flow Cytometric Analysis and Cell Sorting

Lymphocytes were stained with the following anti-human antibodies: anti-CD4 (OKT4), anti-CD49d (9F10), anti-CD44 (IM7), anti-CD20 (L27), anti-CD27 (M-T271), anti-CD43 (MEM-59), anti-CD62L (DREG-56), anti-CXCR5 (J252D4), anti-PD-1 (EH12.2H7), anti-CCR7 (G043H7), anti-CXCR3 (G025H7), anti-CD56 (5.1H11), and anti-ICOS (C398.4A) (BioLegend, San Diego, CA, USA). Flow cytometric analyses were performed using a BD FACS Canto II flow cytometer (BD Biosciences, San Jose, CA, USA). Both CD49d^high^ and CD49d^low^ CD4^+^ T cells were sorted using a BD FACS Aria II (BD Biosciences) after staining with anti-CD4 and anti-CD49d antibodies. Human B cell populations (naïve B cells, CD20^+^CD27^−^CD43^−^; memory B cells, CD20^+^CD27^+^CD43^−^; and B-1 cells, CD20^+^CD27^+^CD43^+^) were sorted after staining with anti-CD20, anti-CD27, and anti-CD43 (3). The purities of the sorted cell populations were routinely ≥98%.

### Intracellular Cytokine Staining and Proliferation Assay

Isolated cells were restimulated with PMA (50 ng/ml) and ionomycin (1 mM) for 4 h for intracellular cytokine staining and then were stained with anti-TNF-α (MAb11), anti-interleukin (IL)-2 (MQ1-17H12), anti-IL-17 (BL168), anti-IL-21 (3A3-N2), anti-interferon (IFN)-γ (4S.B3), anti-IL-4 (MP4-25D2), and anti-IL-10 (JES3-9D7) (BioLegend). Next, CD49d^high^ and CD49d^low^ cells (1 × 10^5^) isolated from the PEC or PB were labeled with 10 µM Cell Trace Violet (Invitrogen, Carlsbad, CA, USA) and were activated with 1 µg/ml plate-bound anti-CD3 (OKT3) and anti-CD28 (CD28.2) (BioLegend) for 72 h. Proliferation was measured using a Violet dilution technique coupled with flow cytometric analysis.

### Real-Time PCR

RNA was isolated from sorted cells using TRIzol reagent (Thermo Fisher Scientific, Waltham, MA, USA) and converted to cDNA using a high capacity cDNA reverse transcription kit (Applied Biosystems, Foster City, CA, USA) according to the manufacturer’s specifications. Each reaction mixture consisted of 2× SYBR Green PCR Master Mix (Applied Biosystems) and 10 pmol/μl of primer (Table S1 in Supplementary Material). Real-time RT-PCR analysis was performed on a Prism 7300 (Applied Biosystems).

### *In Vitro* Co-Culture of B Cells and CD4^+^ T Cells and Enzyme-Linked Immunosorbent Assay (ELISA)

Each sorted B cell population (1 × 10^5^ cells/well) was co-cultured with CD49^high^ CD4^+^ T cells or CD49d^low^ CD4^+^ T cells (5 × 10^4^ cells/well) for 5 days in anti-CD3 (OKT3)-bound plates. The levels of IgM and IgG were determined by ELISA. Briefly, 96-well plates were coated with purified anti-IgM or anti-IgG (Bethyl Laboratories, Montgomery, TX, USA), and binding was revealed using horseradish peroxidase-conjugated anti-IgM and anti-IgG (Bethyl Laboratories). Plates were developed with tetramethylbenzidine (TMB) (Thermo Fisher Scientific), and absorbance was measured at a wavelength of 450 nm using a VersaMax microplate reader (Molecular Devices, Sunnyvale, CA, USA).

### Immunofluorescence Microscopy

Doublet CD49d^high^ CD4^+^ T cells in the PB were sorted and cyto-centrifuged at 400 × *g* for 5 min onto silane-coated glass slides. The images were acquired using a Leica TCS Sp8 confocal laser scanning microscope and exported through LAS AF lite (Leica Biosystem, Wetzlar, Germany).

### Measurement of Anti-Blood Group A Antibody Titers

For measurement of human blood group A-specific IgM and IgG, gel card titration methods were used with serial dilution (ID-System DiaMed, Bio-Rad, Hercules, CA, USA) (17). Gel cards were incubated at room temperature for IgM or at 37°C for IgG according to the manufacturer’s instructions.

### Statistical Analysis

All data are shown as the mean ± SEM. Continuous variables were analyzed using Student’s *t-*tests. Correlations were analyzed using Pearson’s correlation analysis. A *P*-value less than 0.05 was considered statistically significant. All analyses were performed using SPSS (version 20.0, IBM Corporation, Armonk, NY, USA).

## Results

### CD49d^high^ CD4^+^ T Cells Are More Prevalent in the Human PEC Than in the SP or PB

**Figure 1:**
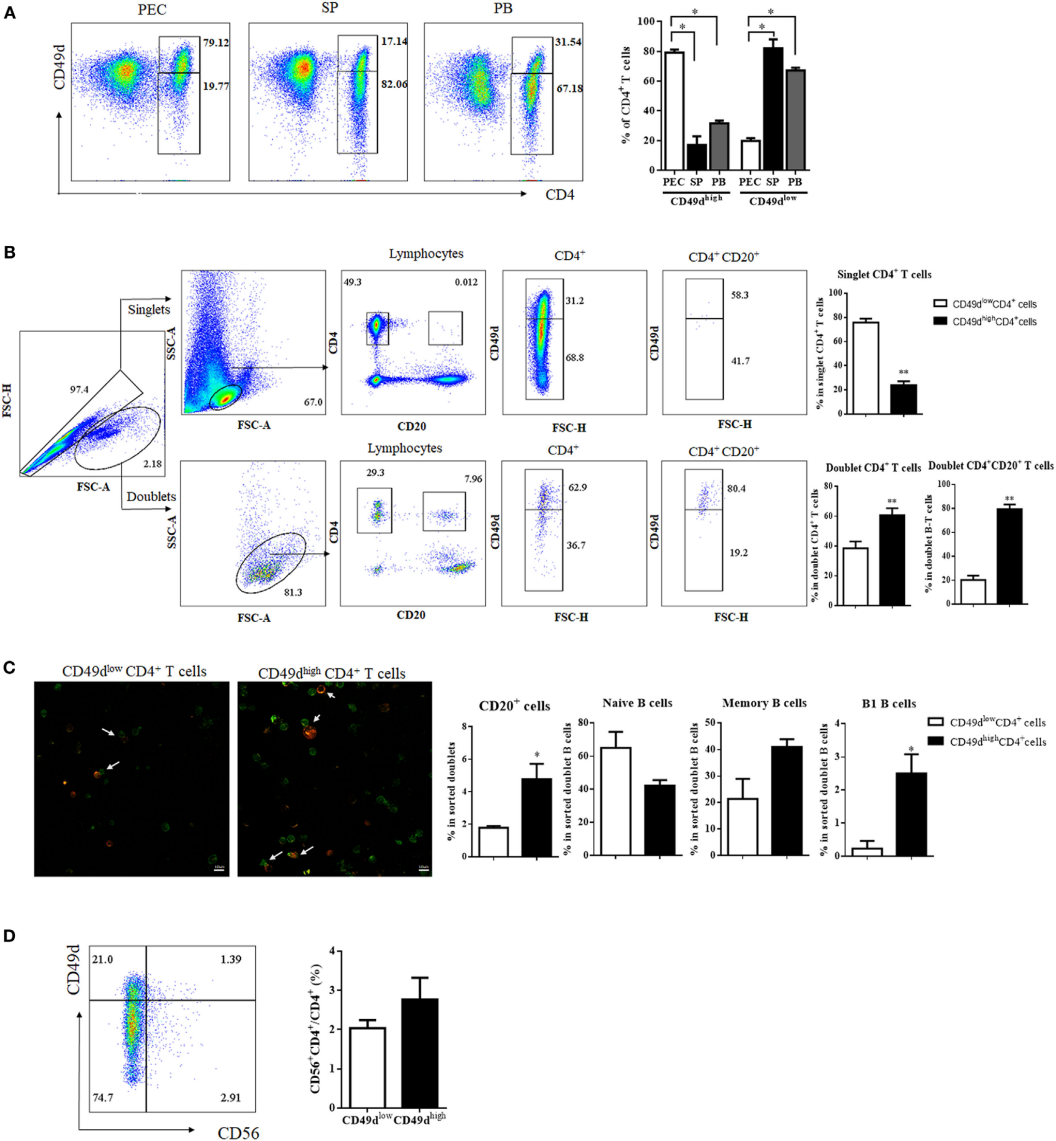
CD49d^high^ CD4^+^ T cells were more prevalent in the human PEC than in the SP or PB. **(A)** Flow cytometric plots showing expression of CD4 and CD49d (integrin α4) in cells from the PEC, SP, and PB. Values in diagrams indicate proportions of the gated population (left panel). Proportions of CD49d^high^ CD4^+^ and CD49d^low^ CD4^+^ T cells among CD4^+^ T cells in the PEC (white), SP (black), and blood (gray) (right panel). **(B)** For analysis of doublet CD4^+^ T cells, singlets and doublets were gated based on FSC-A and FSC-H. After gating for CD4^+^CD20^−^ or CD4^+^CD20^+^, each population was examined for CD49d expression. Values in diagrams indicate proportions of the gated population. Proportions of CD49d^high^ CD4^+^ (black) and CD49d^low^ CD4^+^ T cells (white) in the singlet CD4^+^, doublet CD4^+^, and doublet CD4^+^CD20^+^ cells (right panel). **(C)** Immunofluorescence images of PB B–T conjugates (green: CD4, red: CD20, magnification 200×). White arrows indicate conjugate of B and T cells (left panel). Proportions of total CD20^+^ B cells, CD20^+^ CD27^−^ CD43^−^ naïve B cells, CD20^+^ CD27^+^ CD43^−^ memory B cells, and CD20^+^ CD27^+^ CD43^+^ B-1 cells in CD49d^high^ CD4^+^ (black) and CD49d^low^ CD4^+^ T cell populations (white) (right panel). **(D)** To check for the presence of NKT cells in CD49d^high^ CD4^+^ and CD49d^low^ CD4^+^ T cells, CD4^+^ T cells were examined for the expression of CD56 as well as CD49d. Proportions of CD56^+^ NKT cells among CD49d^high^ CD4^+^ (white) and CD49d^low^ CD4^+^ T cells (black). The data are expressed as the mean ± SEM (*n* = 4–10 donors per each group). *P < 0.05, **P < 0.01; Student’s *t*-test. Abbreviations: PEC, peritoneal cavity; SP, spleen; PB, peripheral blood.

Since CD49d^high^ CD4^+^ T cells with a Th1-like memory phenotype and B-1 cell helper activity are enriched in the mouse PEC (8), we first examined the frequency of CD49d^high^ CD4^+^ T cells in the peritoneal fluid of peritoneal dialysis patients, SPs of brain-death patients, and the PB of healthy donors to determine whether CD49d^high^ CD4^+^ T cells with a memory phenotype are also abundant in the human PEC. Notably, CD49d^high^ CD4^+^ T cells were predominant in the human PEC and were also present in the SP and PB (Figure 1A). The proportion of CD49d^high^ CD4^+^ T cells among CD4^+^ T cells was the highest in the PEC (79.12 ± 2.04%, PEC; 17.14 ± 5.74%, SP; 31.79 ± 1.84%, PB) (Figure 1A). The proportions of CD49d^high^ CD4^+^ T cells in the PB were similar among samples from peritoneal dialysis patients, brain-death donors, and healthy donors (Figure S1A in Supplementary Material). Similar to mouse CD49d^high^ CD4^+^ T cells that interact with peritoneal B-1 cells, human CD49d^high^ CD4^+^ T cells were more frequently found in B-T cell conjugates than as singlet CD4^+^ T cells (Figures 1B,C). When CD49d^high^ CD4^+^ doublets or CD49d^low^ CD4^+^ doublets were analyzed, frequency of CD20^+^ B cells was significantly higher in the doublets with CD49d^high^ CD4^+^ T cells than in those with CD49d^low^ CD4^+^ T cells, especially the frequency of CD27^+^CD43^+^ B-1 cells was higher in the doublets with CD49d^high^ CD4^+^ T cells (Figure 1C). Most CD49d^high^ CD4^+^ T cells were not NKT cells, as both the CD49d^high^ CD4^+^ and CD49d^low^ CD4^+^ T cell populations were comprised of <3% CD56^+^ NKT cells (2.77 ± 0.55 and 2.04 ± 0.20% for CD49d^high^ and CD49d^low^ CD4^+^ T cells, respectively) (Figure 1D). To exclude the possibility that contaminated NKT cells influenced the functional assays of CD49d^high^ CD4^+^ T cells, we used CD56^−^ CD49d^high^ CD4^+^ T cells for all subsequent functional analyses.

### CD49d^high^ CD4^+^ T Cells Show a Th1-Like Memory Phenotype

**Figure 2:**
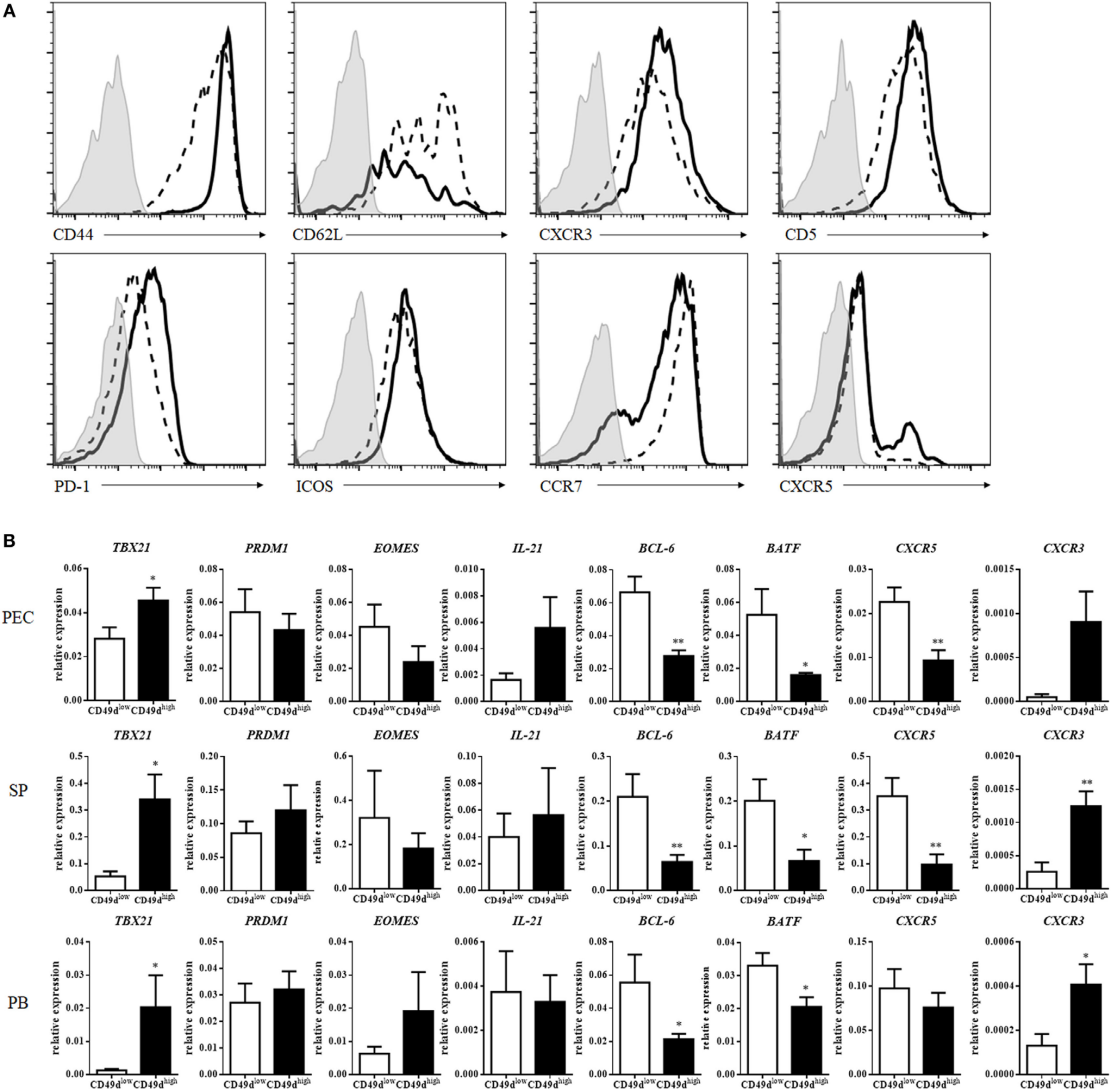
CD49d^high^ CD4^+^ T cells exhibited a Th1-like memory phenotype. **(A)** Expression of surface proteins on human peritoneal CD49d^high^ CD4^+^ T cells (solid line), CD49d^low^ CD4^+^ T cells (dotted line), and isotype control group (gray filled). These data were representative of five independent donor experiments. **(B)** mRNA expression profiles of CD49d^high^ CD4^+^ (black) and CD49d^low^ CD4^+^ T cells (white) in the human PEC, SP, and PB. Real-time analysis was performed using sorted CD49d^high^ CD4^+^ and CD49d^low^ CD4^+^ T cells. The expression levels of various genes in CD49d^high^ CD4^+^ T cells were compared with those in CD49d^low^ CD4^+^ T cells. The data were expressed as the mean ± SEM (*n* = 4–10 donors per each group). *P < 0.05, **P < 0.01; Student’s *t*-test. Abbreviations: PEC, peritoneal cavity; SP, spleen; PB, peripheral blood.

CD49d (integrin α4) can heterodimerize only with integrin β1 or β7 to form VLA4 (integrin α4β1) or LPAM-1 (integrin α4β7), enabling the entrance into inflammatory sites or the mucosa, respectively (18, 19). CD49d^high^ CD4^+^ T cells exhibited much higher expression of integrin β1 than of integrin β7, except in a minor subpopulation of CD49d^high^ CD4^+^ T cells, suggesting that CD49d^high^ CD4^+^ T cells express VLA4 at a high level (Figure S2A in Supplementary Material). CD49d^high^ β1^high^ CD4^+^ T cells also expressed integrin α6 at a high level (Figure S2A in Supplementary Material). In addition to VLA4 and integrin α6, CD49d^high^ CD4^+^ T cells exhibited higher expression levels of CD44, CXCR3, PD-1, ICOS-1, and CD5 and lower levels of CD62L and CCR7 than CD49d^low^ CD4^+^ T cells (Figure 2A). These results indicate that human CD49d^high^ CD4^+^ T cells possess a Th1-like and memory phenotype. However, the expression of CXCR5 was not significantly different between CD49d^high^ and CD49d^low^ CD4^+^ T cells, although a small population of CD49d^high^ CD4^+^ T cells expressed high levels of CXCR5 in some individuals (Figure 2A). The expression level of cell surface molecules in CD49d^high^ CD4^+^ T cells in the PB was similar among samples from peritoneal dialysis patients, brain-death donors, and healthy donors (Figures S1B–D in Supplementary Material). Expression levels of CD44, PD-1, ICOS, and CCR7 were similar between CD49d^high^ β1^high^ β7^−^ CD4^+^ T cells and CD49d^high^ β1^+^ β7^+^ CD4^+^ T cells (Figure S2B in Supplementary Material).

When the mRNA expression levels of Th1-, Th2-, Th17-, and follicular helper T (Tfh)-related molecules were compared between CD49d^high^ CD4^+^ T cells and CD49d^low^ CD4^+^ T cells, the expression of *TBX21*, the gene encoding T-bet for Th1 cell development, was significantly higher in CD49d^high^ CD4^+^ T cells than in CD49d^low^ CD4^+^ T cells (Figure 2B). The expression of *CXCR3*, similar to *TBX21* expression for Th1 cells, was also higher in CD49d^high^ CD4^+^ T cells (Figure 2B). However, the expression levels of *BCL6, BATF*, and *CXCR5* were significantly lower in CD49d^high^ CD4^+^ T cells than in CD49d^low^ CD4^+^ T cells. When we compared expression levels of these gene between CXCR5^−^ CD49d^high^ CD4^+^ T cells and CXCR5^+^ CD4^+^ Tfh cells, CXCR5^−^ CD49d^high^ CD4^+^ T cells showed a trend of higher expression of *TBX21, EOMES, CXCR3, and PRDM1* and lower expression of *IL-21, BCL-6*, and *CXCR5* than Tfh cells, except *BCL6* in the circulating Tfh cells (20) (Figure S3 in Supplementary Material). These results indicate that the CD49d^high^ CD4^+^ T cells had a Th1-like, memory phenotype, but were different from Tfh cells (Figure 2B).

**Figure 3:**
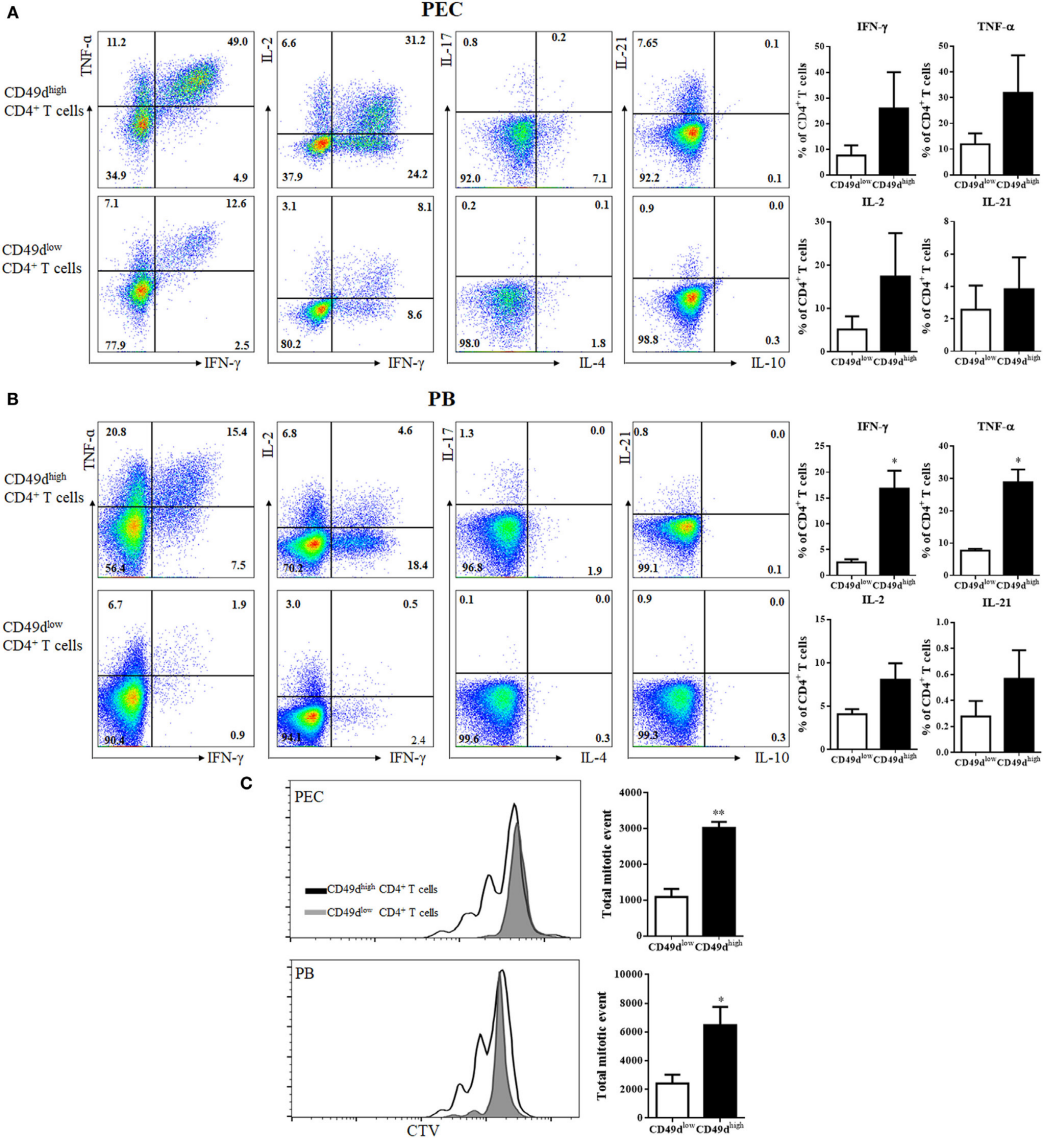
CD49d^high^ CD4^+^ T cells rapidly secreted Th1 cytokines. **(A,B)** Representative flow cytometric plots of intracellular staining for various cytokines (TNF-α, IFN-γ, IL-2, IL-4, IL-10, IL-17, and IL-21). **(A)** Isolated peritoneal cells and **(B)** PB mononuclear cells were stimulated with PMA and ionomycin for 4 h. Cytokine expression in CD49d^high^ CD4^+^ and CD49d^low^ CD4^+^ T cells was analyzed by flow cytometry. Data are representative of three independent experiments (left panel). Proportions of TNF-α, IFN-γ, IL-2, and IL-21 positive CD4^+^ T cells in CD49d^high^ CD4^+^ (black) and CD49d^low^ CD4^+^ T cells (white) in the **(A)** PEC and **(B)** blood (right panel). **(C)** CD49d^high^ CD4^+^ and CD49d^low^ CD4^+^ T cells isolated from the PEC or PB were sorted and labeled with CTV, then stimulated for 72 h with immobilized anti-CD3 and anti-CD28 antibodies. The proliferation of CD49d^high^ CD4^+^ T cells (solid lines) and CD49d^low^ CD4^+^ T cells (gray filled) is shown, along with total mitotic events in CD49d^high^ CD4^+^ (black) and CD49d^low^ CD4^+^ T cells (white). Data are expressed as the mean ± SEM (*n* = 3 donors per each group). *P < 0.05, **P < 0.01; Student’s *t*-test. Abbreviations: TNF-α, tumor necrosis factor-α; IFN-γ, interferon-γ; IL-2, interleukin-2; IL-4, interleukin-4; IL-10, interleukin-10; IL-17, interleukin-17; IL-21, interleukin-21; PMA, phorbol myristate acetate; PEC, peritoneal cavity; PB, peripheral blood.

The CD49d^high^ CD4^+^ T cells were investigated for their ability to secrete various cytokines (Figure 3A). Many peritoneal CD49d^high^ CD4^+^ T cells rapidly secreted IFN-γ (25.96 ± 14.12%), TNF-α (31.92 ± 14.56%), IL-2 (17.38 ± 10.01%), and IL-21 (2.86 ± 2.43%), whereas a much lower proportion of CD49d^low^ CD4^+^ T cells secreted these cytokines (IFN-γ: 7.60 ± 3.94%, TNF-α: 11.94 ± 4.19%, IL-2: 5.17 ± 3.03%, and IL-21: 0.36 ± 0.29%). PB CD49d^high^ CD4^+^ T cells exhibited similar patterns of Th1 cytokine secretion, although a smaller proportion of PB CD49d^high^ CD4^+^ T cells secreted these cytokines compared with the proportion of peritoneal CD49d^high^ CD4^+^ T cells (Figure 3B). When the proliferative capacity of human CD49d^high^ CD4^+^ T cells was compared with that of CD49d^low^ CD4^+^ T cells, both peritoneal and PB CD49d^high^ CD4^+^ T cells exhibited a higher proliferative capacity than CD49d^low^ CD4^+^ T cells (Figure 3C). Taken together, human CD49d^high^ CD4^+^ T cells showed a Th1-like and memory phenotype based on the expression of cell surface molecules and cytokine secretion patterns.

### CD49d^high^ CD4^+^ T Cells in the Umbilical Cord Blood (UCB) Exhibit a Similar Th1-Like and Memory Phenotype

**Figure 4:**
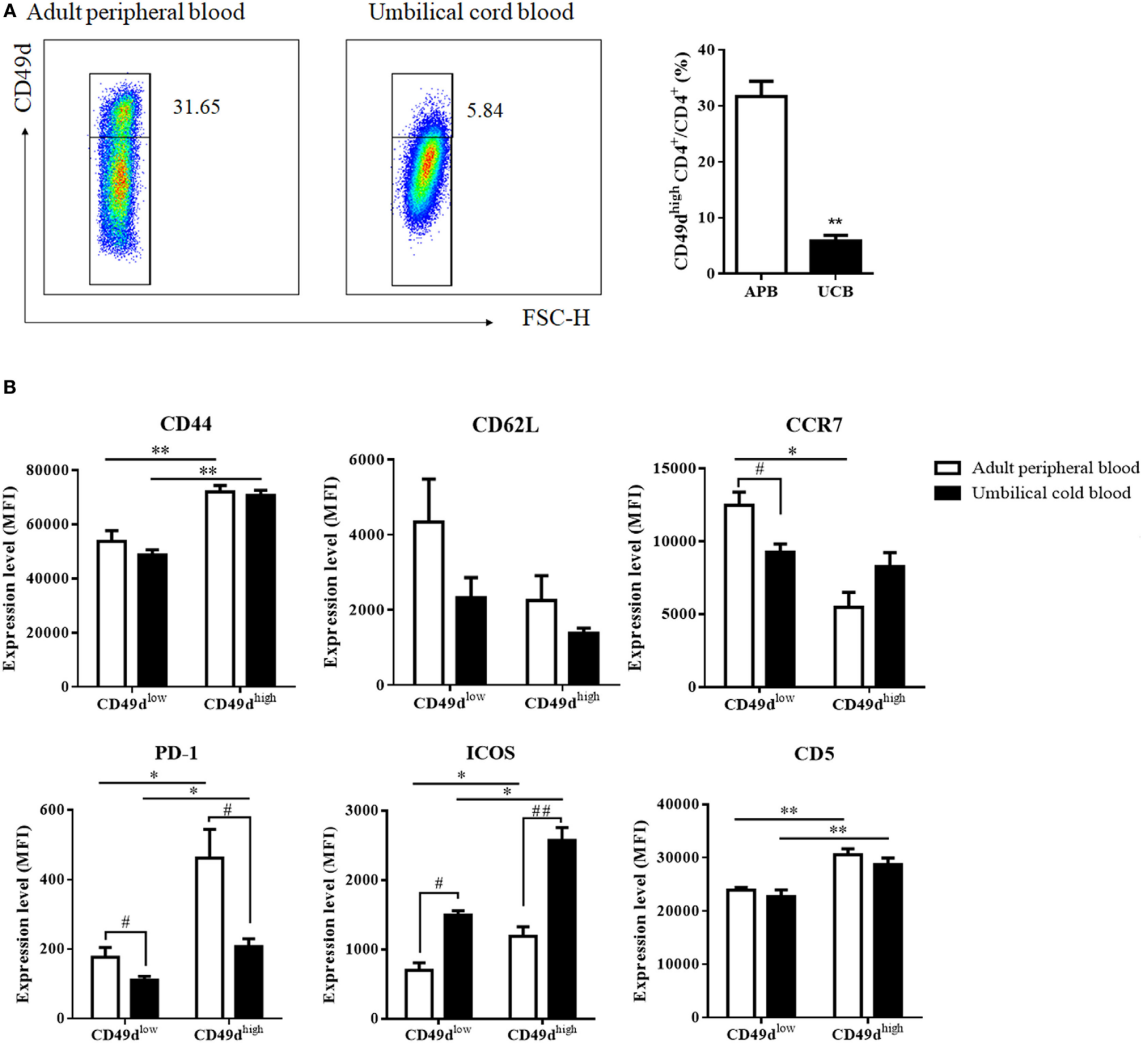
CD49d^high^ CD4^+^ T cells from the umbilical cord exhibit a similar Th1-like memory phenotype. **(A)** CD4^+^ T cells from the PB of healthy adult donors or UCB were analyzed for CD49d expression. Proportions of CD49d^high^ CD4^+^ among CD4^+^ T cells in the APB (white) and the UCB (black) are shown. CD49d^high^ CD4^+^ cells were also identified in the UCB; however, proportions were lower in the UCB than in the APB. Data are representative of three independent experiments. **(B)** When expression levels of various cell surface proteins (CD44, CD62L, CCR7, PD-1, ICOS, and CD5) in CD49d^high^ CD4^+^ T cells were compared between APB and UCB, cell surface phenotypes of CD49d^high^ CD4^+^ T cells in the UCB showed similar patterns as those in APB. Data are expressed as the mean ± SEM (*n* = 3 donors per each group). *P < 0.05, **P < 0.01 in the comparison between CD49d^high^ CD4^+^ T cells and CD49d^low^ CD4^+^ T cells; ^#^*P* < 0.05, ^##^*P* < 0.01 in the comparison between APB and UCB; Student’s *t*-test. Abbreviations: APB, adult peripheral blood; UCB, umbilical cord blood; MFI, mean fluorescence intensity; PB, peripheral blood.

Since mouse CD49d^high^ CD4^+^ T cells are thought to be innate-like lymphocytes capable of developing as early as 3 days after thymectomy, we investigated whether newborns possess CD49d^high^ CD4^+^ T cells by checking for these cells in the UCB. The proportion of CD49d^high^ CD4^+^ T cells was lower in the UCB (5.84 ± 1.03%) than in the adult blood (Figure 4A). However, CD49d^high^ CD4^+^ T cells were clearly present, with cord blood CD49d^high^ CD4^+^ T cells exhibiting higher expression levels of CD44, PD-1, ICOS-1, and CD5 and lower expression levels of CD62L and CCR7 than CD49d^low^ CD4^+^ T cells, similar to adult PB CD49d^high^ CD4^+^ T cells (Figure 4B). Taken together, these data demonstrated that CD49d^high^ CD4^+^ T cells appear in the early stages of development and exhibit a Th1 memory phenotype.

### CD49d^high^ CD4^+^ T Cells Enhance the Immunoglobulin Secretion by Human B-1 Cells

**Figure 5:**
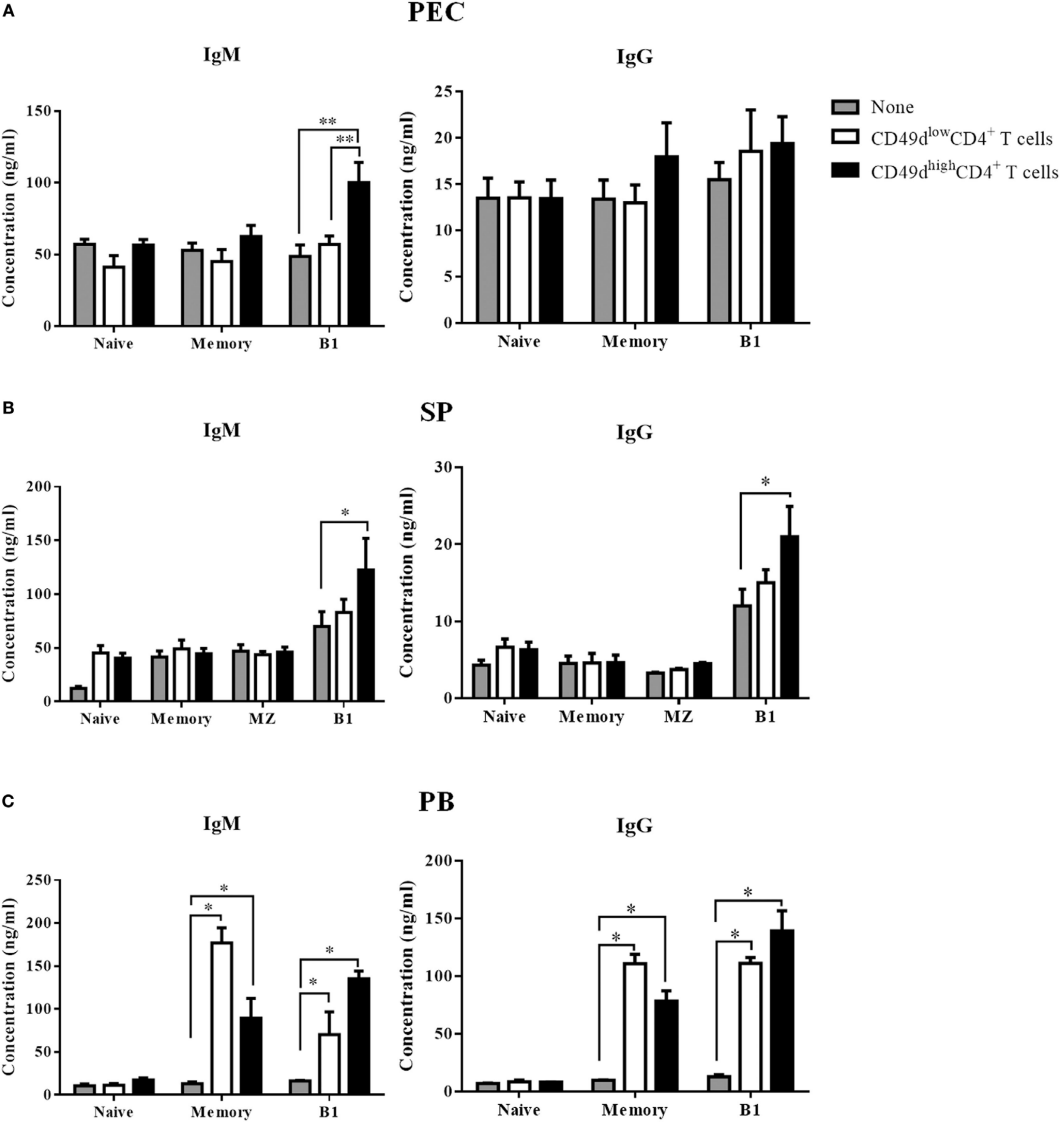
CD49d^high^ CD4^+^ T cells help immunoglobulin secretion by human B-1 cells. **(A)** Sorted peritoneal, **(B)** splenic, and **(C)** PB-derived CD49d^high^ CD4^+^ T cells or CD49d^low^ CD4^+^ T cells were co-cultured with sorted naïve (CD20^+^ CD27^−^CD43^−^ CD1c^−^), memory (CD20^+^ CD27^+^ CD43^−^ CD1c^−^), marginal zone (CD20^+^ CD27^+^ CD43^−^ CD1c^+^), and B-1 (CD20^+^ CD27^+^ CD43^+^ CD1c^−^) B cells for 5 days in the presence of immobilized anti-CD3 antibody. Concentrations of human IgM and IgG in the culture supernatant were compared among the B cell alone group (gray), the CD49d^high^ CD4^+^ T cell group (black), and the CD49d^low^ CD4^+^ T cell group (white). Data are expressed as the mean ± SEM (*n* = 3–5 donors per each group). *P < 0.05, **P < 0.01; Student’s *t*-test. Abbreviations: PEC, peritoneal cavity; SP, spleen; PB, peripheral blood.

The PEC was enriched in CD49d^high^ CD4^+^ T cells, but lacked B cells. Although the presence of human B-1 cells has been questionable since CD5 was shown not to be a reliable marker for human B-1 cells (21), the identity of human B-1 cells has been reported since several years ago (3, 7). Therefore, we investigated whether CD49d^high^ CD4^+^ T cells could help B-1 cells secrete antibodies. We sorted naïve (CD20^+^CD27^−^CD43^−^CD1c^−^), memory (CD20^+^CD27^+^CD43^−^CD1c^−^), marginal zone (CD20^+^CD27^+^CD43^−^CD1c^+^), and B-1 (CD20^+^CD27^+^CD43^+^CD1c^−^) B cells from the PB and SP samples (Figure S4 in Supplementary Material). Both CD56^−^ CD49d^high^ CD4^+^ T cells and CD56^−^ CD49d^low^ CD4^+^ T cells were also sorted from the PB, SP, and peritoneal fluids. After 5 days of co-culturing individual CD4^+^ T and B cell subsets, both IgM and IgG titers were measured in the supernatants. Co-culture with CD49d^high^ CD4^+^ T cells from the peritoneal fluid (Figure 5A), PB (Figure 5B), and SP (Figure 5C) increased IgM secretion from B-1 cells, whereas co-culture with CD49d^low^ CD4^+^ T cells did not. CD49d^high^ CD4^+^ T cells also helped B-1 cells secrete IgG (Figures 5B,C). While CD49d^low^ CD4^+^ T cells increased the secretion of both IgM and IgG from memory B cells in the PB (Figure 5B), neither CD49d^high^ CD4^+^ nor CD49d^low^ CD4^+^ T cells promoted immunoglobulin secretion by splenic marginal zone B cells (Figure 5C). When we sorted CXCR5^−^CD49^high^ or CD49^low^ CD4^+^ T cells to exclude CXCR5^+^ Tfh cells, the CXCR5^−^CD49^high^ CD4^+^ T cells, but not Tfh cells, could still help immunoglobulin secretion from B-1 cells (Figure S5 in Supplementary Material). Taken together, human CD49d^high^ CD4^+^ T cells in the peritoneal fluid, PB, and SP specifically enhanced the immunoglobulin secretion by human B-1 cells.

### Correlation Between Frequency of CD49d^high^ CD4^+^ T Cells and Serum Levels of Anti-Glycan IgM Secretion

**Figure 6:**
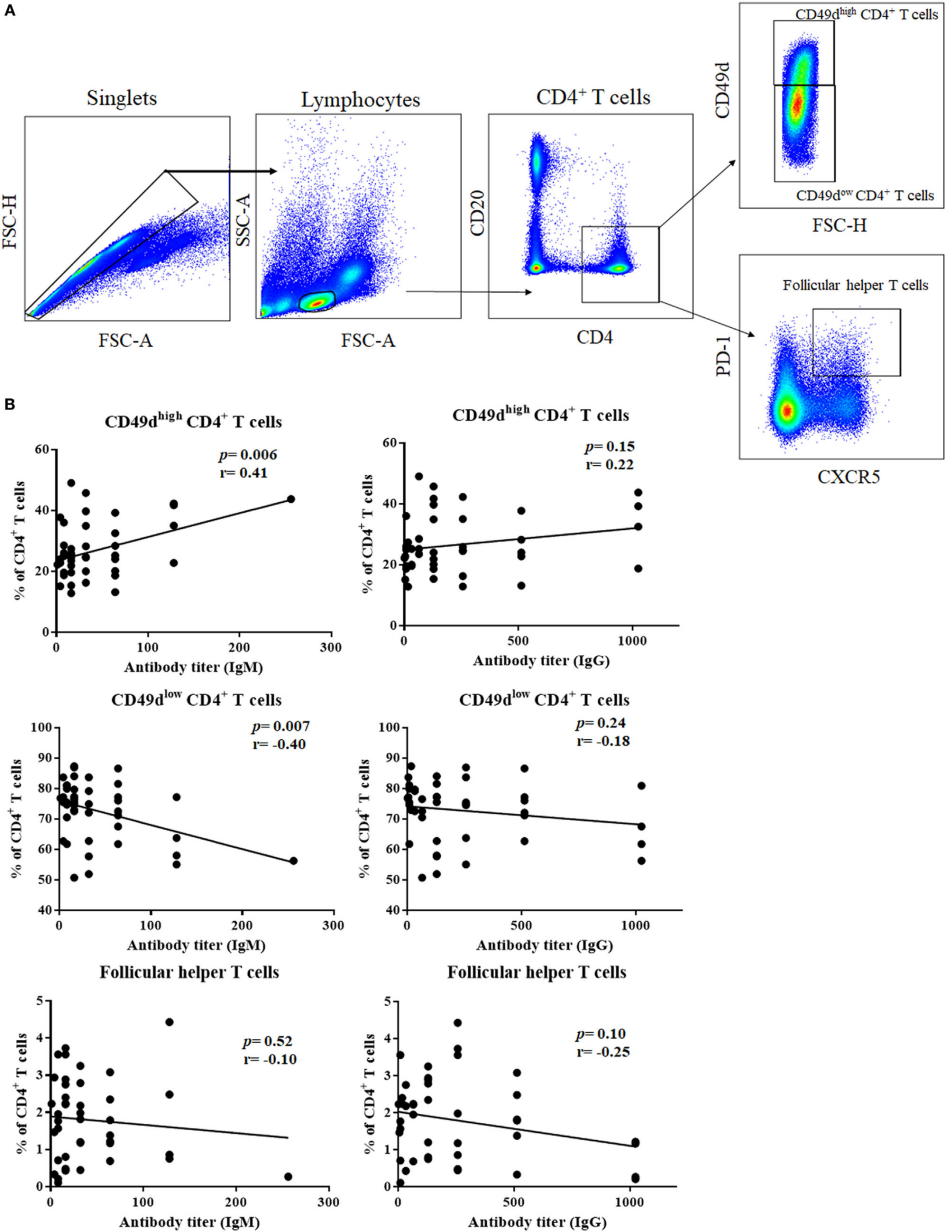
Frequency of CD49d^high^ CD4^+^ T cells were positively correlated with anti-human blood group A antibody titer. **(A)** Follicular helper T cells, CD49d^high^ CD4^+^ T cells, and CD49d^low^ CD4^+^ T cells were gated from human peripheral blood lymphocytes using CD4, CD49d, CXCR5, and PD-1. **(B)** Correlations between the frequency of each population and anti-human blood group A IgM/IgG titers are shown. Anti-A IgM titer was positively associated with the frequency of CD49d^high^ CD4^+^ T cells (*P* = 0.0059; *r* = 0.4083) and negatively correlated with the frequency of CD49d^low^ CD4^+^ T cells (*P* = 0.0069; *r* = −0.4017). However, neither CD49d^high^ CD4^+^ T cells nor CD49d^low^ CD4^+^ T cell frequencies correlated with anti-A IgG titer. There was no correlation between the frequency of follicular helper T cells and anti-A antibody titer. Correlations were analyzed by Pearson’s correlation analysis.

The ability of CD49d^high^ CD4^+^ T cells to help B-1 cells led us to investigate whether CD49d^high^ CD4^+^ T cells are linked to the production of antibodies against T cell-independent antigens, such as glycan. We investigated the correlation between the titer of antibody against blood group A antigen and the frequencies of CD49d^high^ CD4^+^ T cells or Tfh cells in the PB (Figure 6A). The titer of anti-A IgM was positively correlated with the frequency of CD49d^high^ CD4^+^ T cells (*P* = 0.0059, *r* = 0.4083, Figure 6B), and anti-A IgM titer was negatively correlated with that of CD49d^low^ CD4^+^ T cells (*P* = 0.0069, *r* = −0.4017, Figure 6B). However, there was no significant correlation between the anti-A IgG titer and CD49d^high^ CD4^+^ T cells (Figure 6B). Furthermore, we found no significant correlation between the anti-A titer and the frequency of follicular helper T cells (Figure 6B). These results suggest that CD49d^high^ CD4^+^ T cells specifically help B-1 cells secrete antibodies and are distinct from Tfh cells.

## Discussion

In this study, we report for the first time that human CD49d^high^ CD4^+^ T cells in the PEC and blood exhibit a Th1-like memory phenotype and a unique capacity to help B-1 cells produce natural antibodies. Generally, B-1 cells are thought to function in a manner independent of CD4^+^ T cells. However, evidence showing that B-1 cells are excellent antigen-presenting cells (6, 7) also suggest the possibility that B-1 cells may be activated by CD4^+^ T cells *via* a mutual interaction (8, 9). We observed a high frequency of conjugates between human CD49d^high^ CD4^+^ T and B-1 cells, indicating the close interaction of these populations.

The majority of CD49d^high^ CD4^+^ T cells expressed high levels of integrin β1 to form VLA4. The expression of VLA4 appears to be carefully regulated, since high expression of VLA4 enables VLA4^+^ cells to enter inflammatory sites, such as the draining lymph nodes and brain (19, 22). The blockade of CD49d, and thus VLA4, is currently used as a treatment to inhibit neuroinflammation in multiple sclerosis (22). In the SP and blood, most memory T cells do not express a high level of CD49d, and only a small portion of CD4^+^ memory T cells expressing a high level of CD49d also characteristically express PD-1, ICOS, and CD5, indicating their prolonged activation status. These cells rapidly proliferated and produced IFN-γ, TNF-α, and IL-2 upon stimulation. Furthermore, the expression of both VLA-4 and VLA-6 (α6β1) in most of the CD49d^high^ CD4^+^ T cells suggest their potential of transendothelial and interstitial migration into inflamed tissues *via* respective integrin receptors. Interestingly, this feature is shared in human and mouse CD49d^high^ CD4^+^ T cells (23).

Remarkably, CD49d^high^ CD4^+^ T cells specifically helped B-1 cells, but not for follicular or marginal zone B cells, in increasing the production of IgM and IgG antibodies. This kind of B cell help is a unique form that differs from previously described types of B cell help (24). The classical pathway of B cell help is through the CD40–CD40L interaction, by inducing B cell proliferation. Other types of B cell help include effects on B cell survival, plasma cell differentiation, induction of somatic hypermutation, and class switching recombination. CD49d^high^ CD4^+^ T cells induced B-1 cells to secrete more antibodies for several days. Although B-1 cells are not fully differentiated plasma cells, they exhibit some features of plasma cells, such as the expression of Blimp-1 (25). To date, how B-1 cells regulate the production of natural antibodies is not well understood. It is likely that B-1 cells produce more antibodies in the face of infection or injury, although B-1 cells appear to produce antibodies constitutively when examined over a long period. In fact, long-lived plasma cells have been shown to produce antibodies upon inflammatory signals and interactions with bone marrow dendritic cells (26). Thus far, CD49d^high^ CD4^+^ T cells are the first T cell population shown to enhance the antibody secretion of co-cultured B cells in both the mouse and human systems. Our data showed that this kind of B cell help is highly specific for B-1 cells and is not observed in memory B cells or marginal zone B cells. An interesting question is why CD49d^high^ CD4^+^ T cells do not induce antibody secretion by marginal zone B cells. We assume that marginal zone B cells are pre-activated cells, but in contrast to B-1 cells, they are not prepared for immediate antibody secretion. At the same time, the human anti-blood group A IgM titer was positively correlated with the frequency of CD49d^high^ CD4^+^ T cells. As anti-A antibody has been shown to be generated by B-1 cells (27), this association consolidates the identity of the CD49d^high^ CD4^+^ T cells as B-1 helper T cells. Corresponding to the previous notion that B-1 cells do not undergo germinal center reactions, we found that there was no correlation between the frequency of follicular helper T cells and the anti-A titer (28, 29).

The CD49d^high^ CD4^+^ T cells were quite different from classical follicular helper T cells but were rather a kind of innate-like Th1 cell. Th1 cells were previously shown to play a significant role in the generation of protective antibodies against the influenza virus, even with the genetic ablation of follicular helper T cells (30), suggesting that Th1 cells can function as B helper T cells for short-term antibody production. Notably, Tfh cells could not help immunoglobulin secretion from all kinds of B cells under our experimental condition. We interpret that Tfh cells take a long time (>6 days) to help B cells produce immunoglobulin since Tfh cells basically promote B cell proliferation and differentiation into germinal center B cells and antibody secretion is noted about more than 6 days after co-culture of Tfh and B cells (31). By contrast, CD49d^high^ CD4^+^ T cells is thought to stimulate B cell to secrete antibody rapidly and thus to be a different type of B helper T cells. These characteristics are similar to previously described ICOS-dependent extrafollicular helper T cells that enhance IgG secretion 96 h after co-culture (32). We speculate that the CD49d^high^ CD4^+^ T cells are a unique type of B-1 helper T cell. At the moment, we do not know how CD49d^high^ CD4^+^ T cells recognize antigens presented by B-1 cells or how they provide help for B-1 cells. Taken together, CD49d^high^ CD4^+^ T cells are a distinct type of extrafollicular, innate-like Th1 cell with a memory phenotype, and they exhibit a helper function, enhancing immunoglobulin production by innate B-1 cells.

The presence of human CD49d^high^ CD4^+^ T cells in the UCB suggests that this innate-like population is generated early in development, similar to mouse CD49d^high^ CD4^+^ T cells (8). Moreover, the expression patterns of the surface molecules on CD49d^high^ CD4^+^ T cells were similar between the UCB and adult PB. Interestingly, the expression levels of PD-1 and ICOS in the UCB differed from those in the adult blood, indicating the immature status of CD49d^low^ CD4^+^ T cells in the UCB (33, 34).

CD49d^high^ CD4^+^ T cells were present mainly in the peritoneum, but they were found in the human SP and PB more frequently than in the mouse SP and PB. When we compared human CD49d^high^ CD4^+^ T cells from different anatomical sites, each CD49d^high^ CD4^+^ T cell population showed similar expression patterns at the gene and protein levels. However, peritoneal CD49d^high^ CD4^+^ T cells had the highest capacity for Th1 cytokine secretion. Moreover, PB CD49d^high^ CD4^+^ T cells exhibited a helper function, enhancing antibody production by memory B-2 cells. At this point, we do not know whether the peritoneal and PB CD49d^high^ CD4^+^ T cells are the same kinds of T cells with some modification according to the local environment or whether they represent different cell types. In mice that grew under SPF conditions, most of the peritoneal CD49d^high^ CD4^+^ T cells are thought to be innate-like lymphocytes since they appeared even in mice that had underwent thymectomy on day 3. However, upon antigenic challenge events with aging, some *bona fide* memory CD4^+^ T cells are thought to obtain CD49d expression. Therefore, CD49d^high^ CD4^+^ T cells could be mixtures of innate-like cells and *bona fide* memory cells. We presume that the peritoneal CD49d^high^ CD4^+^ T cells are enriched in innate-like cells, whereas blood CD49d^high^ CD4^+^ T cells would include more *bona fide* memory CD4^+^ T cells. That is one potential explanation for why CD49d^high^ CD4^+^ T cells can help class-switched memory B cells in the PB. To address this issue more clearly, it is necessary to identify more definitive and stable markers for B-1 helper T cells.

B-1 cells are regarded as the main anti-ABO antibody-secreting cells (35). This study demonstrated that anti-A antibody titer is positively associated with the frequency of CD49d^high^ CD4^+^ T cells and that CD49d^high^ CD4^+^ T cells help B-1 cells secrete immunoglobulin. Therefore, the CD49d^high^CD4^+^ T cells could be a potential target for developing treatments that suppress anti-ABO antibody titer and subsequent antibody-mediated rejection in ABO-incompatible organ transplantation. Further interventional studies are needed for elucidating clinical roles of CD49d^high^ CD4^+^ T cells.

There are a few limitations of this study. First, the peritoneal samples were obtained from peritoneal dialysis patients, who exhibited an underlying renal disease and a mild degree of chronic peritoneal inflammation (36). The SP samples were obtained from brain-death donors, who are considered to have a mild degree of inflammation (37). However, when we compared proportions and expression of surface molecules of CD49d^high^ CD4^+^ T cells in the PB samples from healthy donors, peritoneal dialysis patients, and brain-death donors, there was no significant difference (Figure S1 in Supplementary Material). Second, CD49d may be not a definitive marker for B-1 helper T cells, since CD49d expression was altered upon the adoptive transfer of mouse CD49d^high^ CD4^+^ T cells into Rag1^−/−^ mice (8). Therefore, further studies are needed for the identification of more reliable markers of B-1 helper T cells.

In conclusion, human CD49d^high^ CD4^+^ T cells that are mainly present in the PEC are innate-like Th1 CD4^+^ T cells with a memory phenotype that can rapidly secrete Th1 proinflammatory cytokines and help B-1 cells produce immunoglobulin, thereby playing a primary defense role in the PEC and other sites.

## Ethics Statement

This study was carried out in accordance with the recommendations of the Declaration of Helsinki. The protocol was approved by the Institutional Review Board of Seoul National University Hospital (H-1411-020-623). All subjects gave written informed consent for sample donation in accordance with the Declaration of Helsinki.

## Author Contributions

J-GL, TJK, and JY contributed conception and design of the study. J-GL, J-HR, TYK, DK, and K-HO prepared samples. J-GL, JJ, TF, YX, and J-JY performed experiments. J-GL and TYK performed the statistical analysis. J-GL wrote the first draft of the manuscript. TJK and JY wrote the final form of the manuscript. All the authors contributed to manuscript revision, read and approved the submitted version.

## Conflict of Interest Statement

The authors declare that the research was conducted in the absence of any commercial or financial relationships that could be construed as a potential conflict of interest.
