# Supplementary material for: Identification of Human B-1 Helper T Cells With a Th1-Like Memory Phenotype and High Integrin CD49d Expression
Source: Front Immunol. 2018 Jul 16;9:1617. doi: 10.3389/fimmu.2018.01617 (PMC6054961; doi:10.3389/fimmu.2018.01617)
Supplement: Supplementary file 1 [file Data_Sheet_1.PDF]

Supplementary Material

Identification of Human B-1 Helper T Cells with a Th1-like Memory Phenotype and High Integrin CD49d Expression

Jae-Ghi Lee, Joon Young Jang, Taishi Fang, Yixuan Xu, Ji-Jing Yan, Jung Hwa Ryu, Hee Jung Jeon, Tai Yeon Koo, Dong Ki Kim, Kook Hwan Oh, Tae Jin Kim, Jaeseok Yang

Correspondence: Jaeseok Yang, jcyjs@dreamwiz.com; Tae Jin Kim, tjkim@skku.edu

Supplementary Table

Table S1. Primer sets used for real-time reverse transcription-polymerase chain reaction

Gene	Primer sequence (5'-3')	Annealing temperature (℃)	Product size (bp)
IL-21	F:CAAATCAAGCTCCCAAGGTCA	60	110
	R:GCAGAAATTCAGGGACCAAG TC		
Batf	F: GTGAGAAGAGTTCAGAGGAGGGAG	60	120
	R: CGCGTTCTGT TTCTCCAGG		
Bcl-6	F: TTCCGGCACCTTCAGACTCT	60	99
	R: GCTTTTGTGACGGAAATGCA		
CXCR5	F: GACACCTCCCTGGTGGAAAA	60	99
	R: GATGAGGCTGTAGGCCACG		
Prdm1	F: CACTGTGAGGTTTCAGGGATTG	60	103
	R: CTCTTCAAAC TCAGCCTCTG TCC		
Tbx21	F: CGCAGCACCGCTACTTCTAC	60	137
	R: GGCGTAGGCT CCAAGGAAG		
Eomes	F: GCTACTCCATGGACAGCCTG	60	138
	R: AGCCGGGTA CACAGGTCC		

F, forward; R, reverse; IL-21, Interleukin 21; Batf, Basic leucine zipper transcription factor ATP-like; Bcl-6, B-cell lymphoma 6 protein; CXCR5, C-X-C chemokine receptor type 5; Prdm1, PR domain zinc finger protein 1; Tbx21, T-box transcription factor TBX21; Eomes, Eomesodermin.

Supplementary Figures

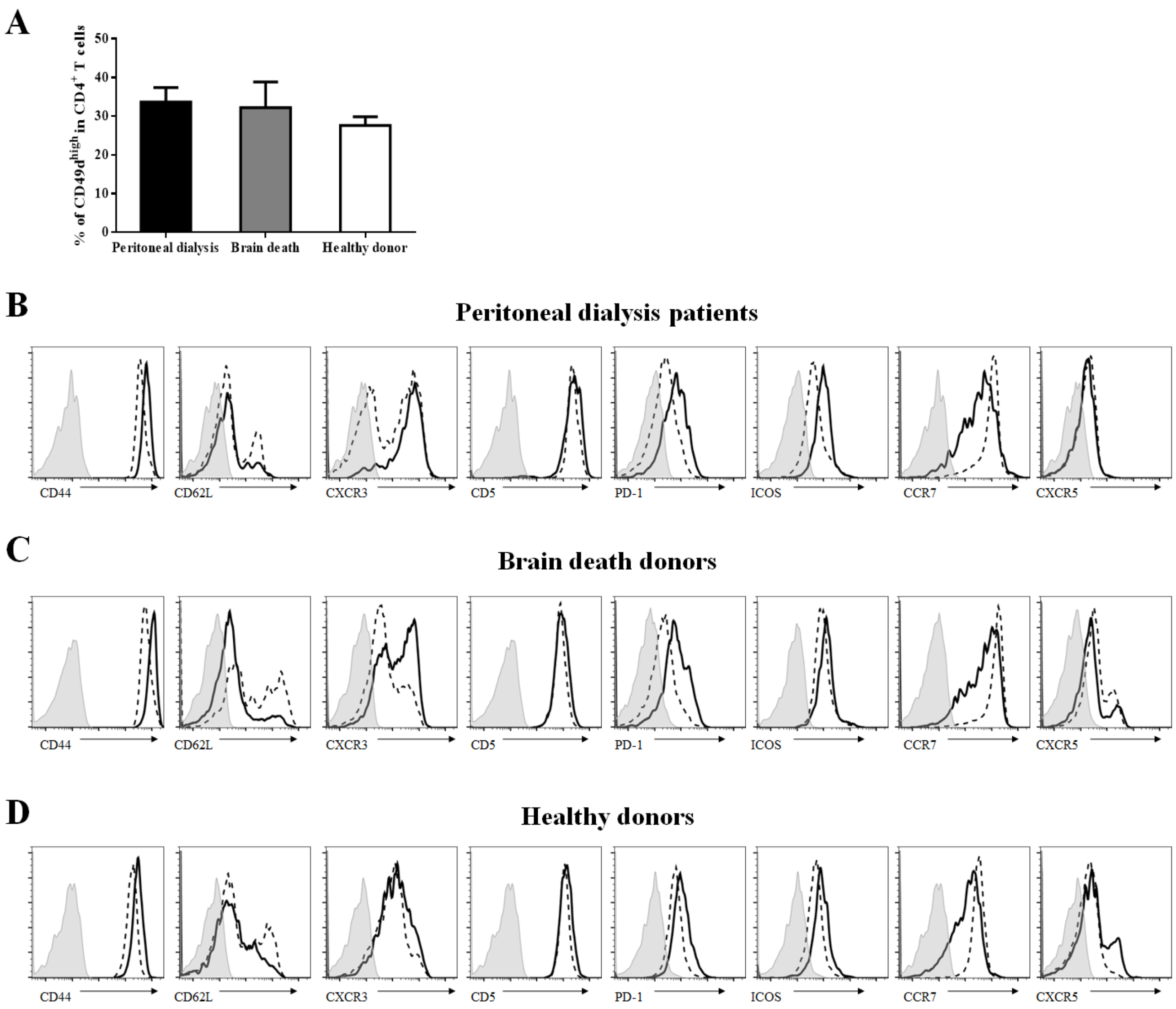

Figure S1. Peripheral blood CD49d^{high} CD4^{+} T cells from peritoneal dialysis patients, brain-death donors, and healthy donors, show similar surface protein expression. (A) Proportions of CD49d^{high} CD4^{+} T cells in the peripheral blood from the peritoneal dialysis patients, brain-death donors, and healthy donors. The data are expressed as the mean ± standard error of the mean (n = 5 donors per each group). *$P < 0.05$; Student's *t*-test. (B-D) Expression of surface proteins on CD49d^{high} CD4^{+} T cells (solid line), CD49d^{low} CD4^{+} T cells (dotted line), and isotype control group (gray filled) from (B) peritoneal dialysis patients, (C) brain-death donors, or (D) healthy donors. These data were representative of five independent donor experiments.

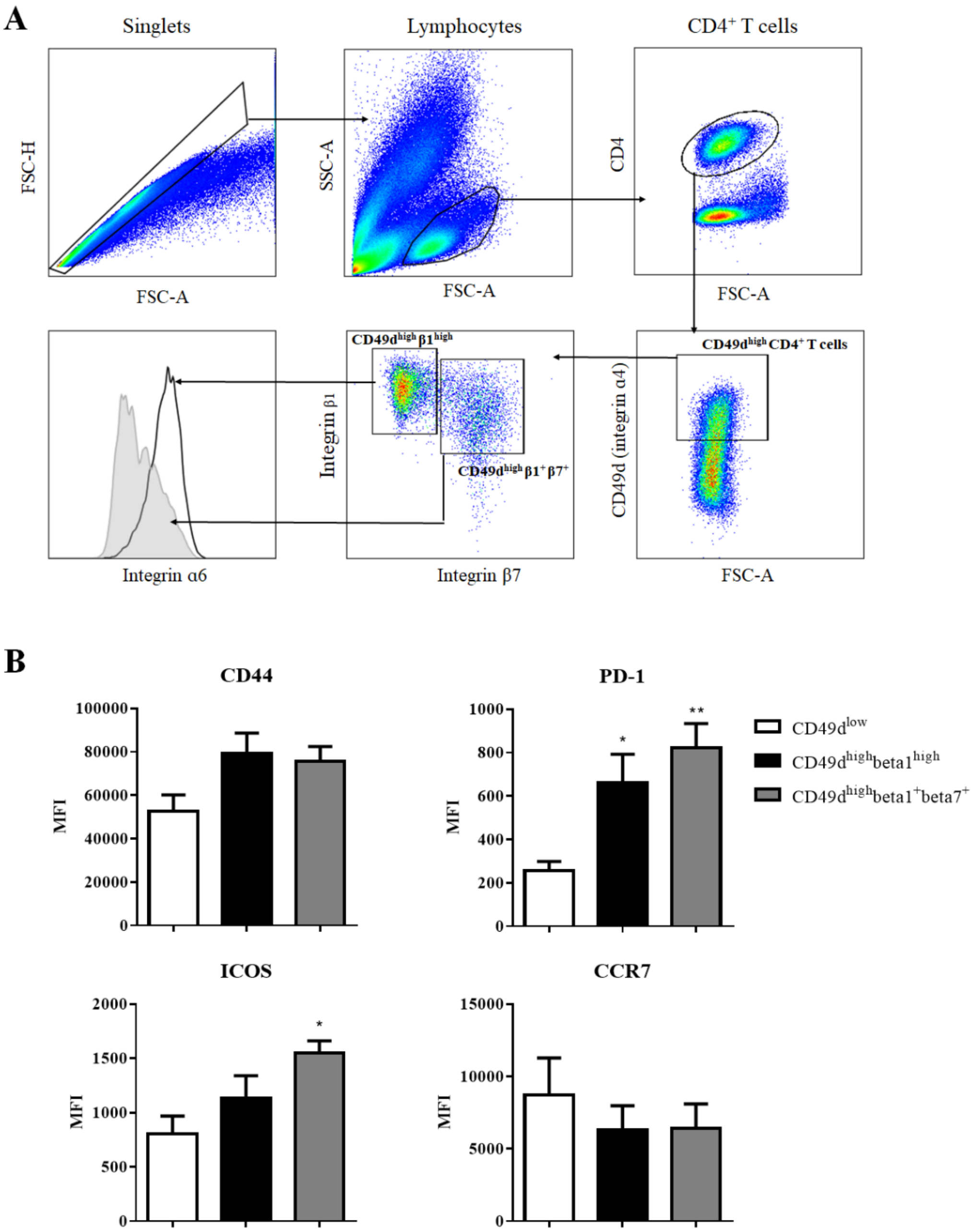

Figure S2. CD49d^{high} CD4^{+} T cells contain integrin α4^{high} β1^{high} T cells and α4^{high} β1^{+} β7^{+} T cells. (A) Peripheral blood CD4^{+} T cells were examined for expression of CD49d (integrin α4), integrin β1, integrin β7, and integrin α6. Next, we compared the expression levels of integrin α6 among CD49d^{high} β1^{high} CD4^{+} (solid line) and CD49d^{high} β1^{+} β7^{+} CD4^{+} T cells (filled gray). (B) We compared expression of surface proteins (CD44, PD-1, ICOS, and CCR7) between CD49d^{high} β1^{high} T cells and CD49d^{high} β1^{+} β7^{+} T cells and found that there was no significant difference between the two cell populations. Data are expressed as the mean ± standard error of the mean (n = 3 donors per group). *P < 0.05, **P < 0.01 in the comparison between CD49d^{high} CD4^{+} and CD49d^{low} CD4^{+} T cells; $^{\#}P$ < 0.05 in the comparison between CD49d^{high} β1^{high} and CD49d^{high} β1^{+} β7^{+} CD4^{+} T cells; Student's *t*-test.

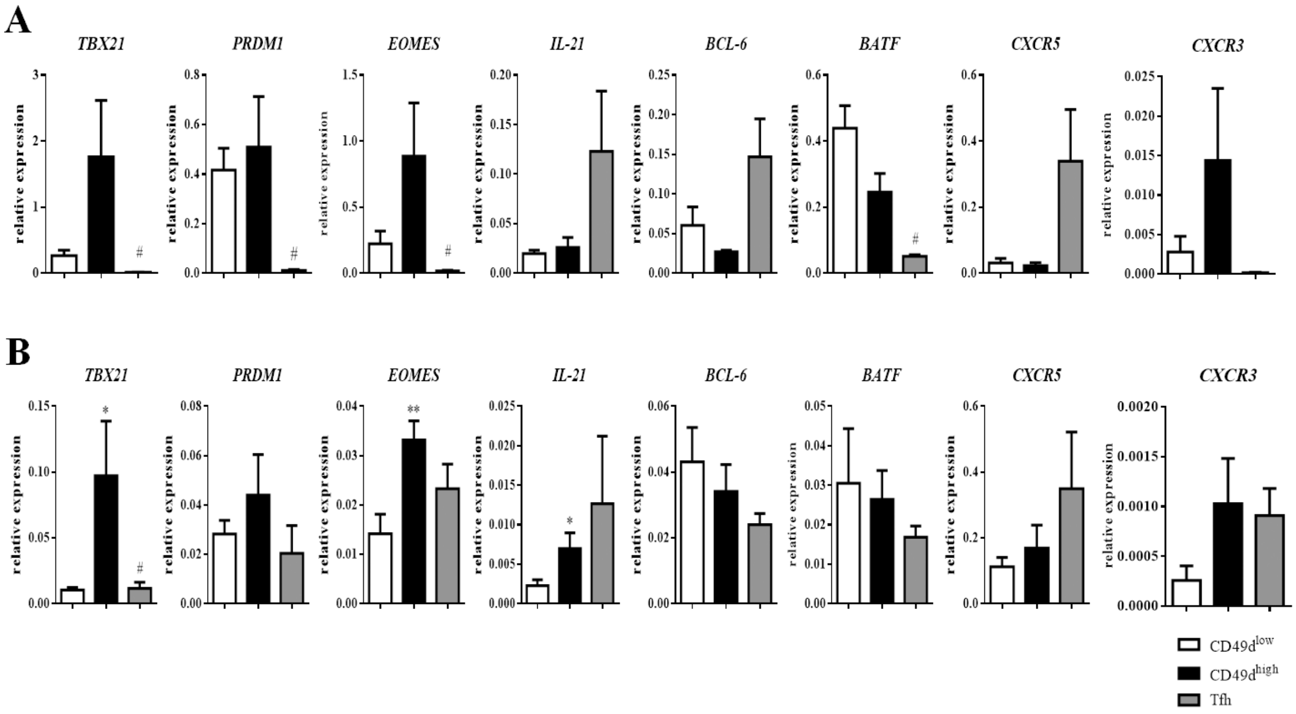

Figure S3. CXCR5$^-$ CD49d^{high} CD4$^+$ T cells that are distinct from follicular helper T cells, exhibit a Th1-like memory phenotype. The mRNA expression profiles of CXCR5$^-$ CD49d^{high} CD4$^+$ (black), CXCR5$^-$ CD49d^{low} CD4$^+$ (white), and CXCR5$^+$ CD4$^+$ follicular helper T cells (gray) in the human spleen (A) and peripheral blood (B). The expression levels of various genes in CXCR5$^-$ CD49d^{high} CD4$^+$ were compared with those in CXCR5$^-$ CD49d^{low} CD4$^+$ T cells and follicular helper T cells. The data were expressed as the mean ± standard error of the mean (n = 3 donors per group). $^*P < 0.05$, $^{**}P < 0.01$ in the comparison between CXCR5$^-$ CD49d^{low} and CXCR5$^-$ CD49d^{high} CD4$^+$ T cells, $^{\#}P < 0.05$, $^{\#\#}P < 0.01$ in the comparison between CXCR5$^-$ CD49d^{high} CD4$^+$ T cells and follicular helper T cells; Student's *t*-test. SP, spleen; PB, peripheral blood; Tfh, follicular helper T cells.

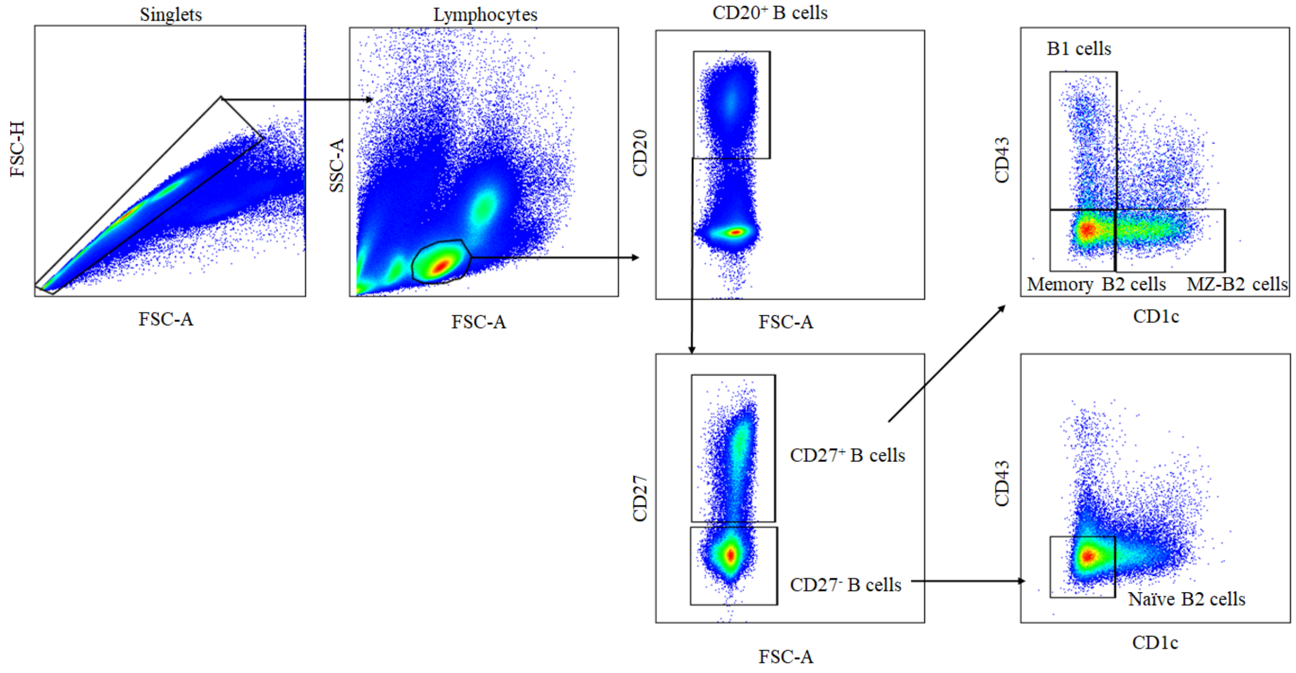

Figure S4. Gating strategy for naïve, memory, marginal zone, and B1 B cells. For analysis of B1 cells in human peripheral blood, singlet blood lymphocytes were gated based on FSC-A and SSC-A. Then, $CD20^+$ B cells were divided into two populations according to CD27 expression. The $CD43^–$ $CD27^–$ B cells were naïve B2 cells. The $CD27^+$ B cells were subdivided into memory B2 cells ($CD27^+CD43^-CD1c^–$), marginal zone B cells ($CD27^+CD43^-CD1c^+$), and B1 cells ($CD20^+CD27^+CD43^+CD1c^–$) based on differential expression of CD43 and CD1c.

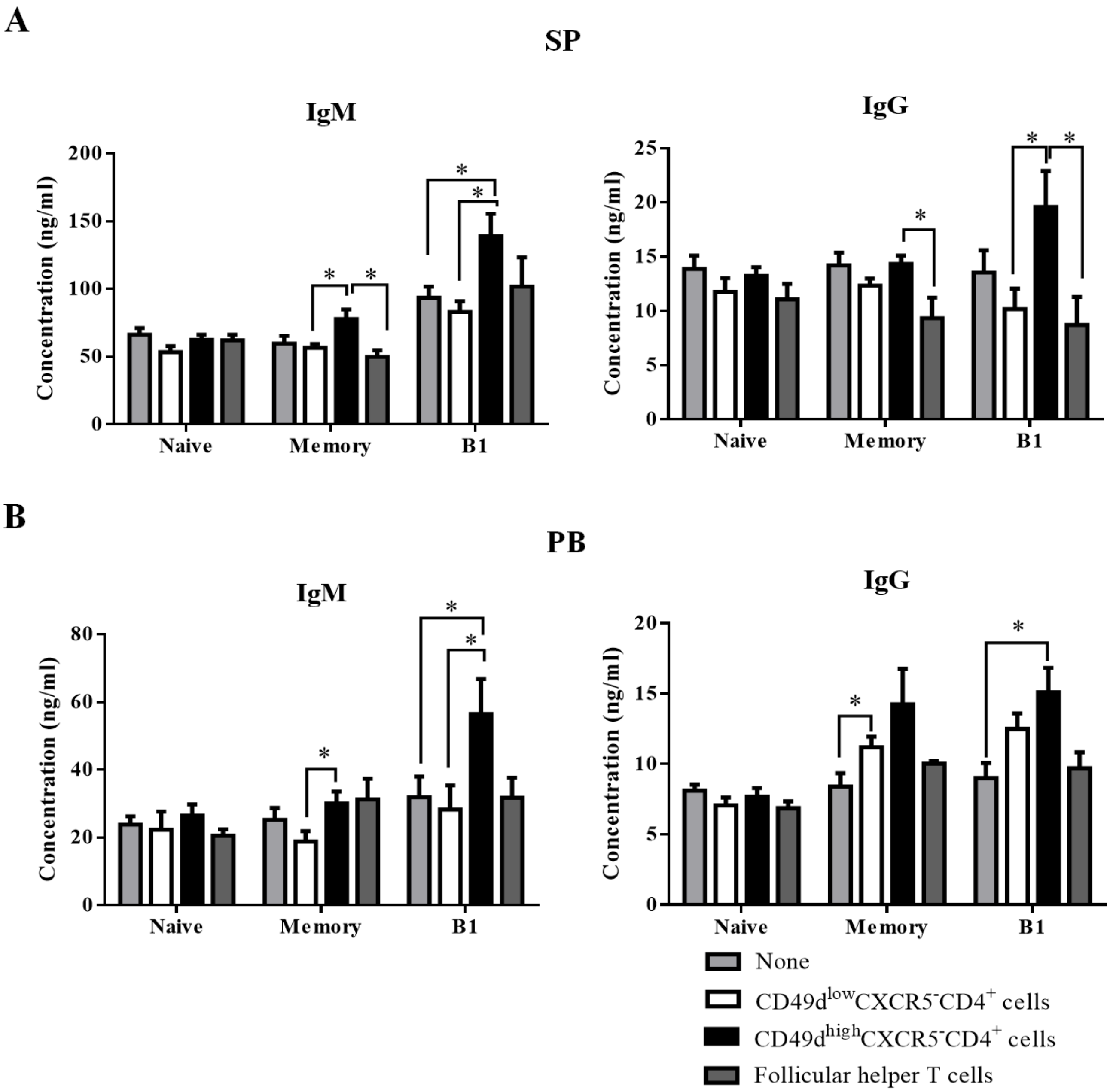

Figure S5. CXCR5$^-$ CD49d^{high} CD4$^+$ T cells help immunoglobulin secretion by human B-1 cells. (A) Sorted splenic and (B) peripheral blood-derived CXCR5$^-$ CD49d^{high} CD4$^+$ T cells, CXCR5$^-$ CD49d^{low} CD4$^+$ T cells, or CXCR5$^+$ CD4$^+$ follicular help T cells were co-cultured with sorted naïve (CD20$^+$ CD27$^-$–CD43$^-$ CD1c$^-$), memory (CD20$^+$ CD27$^+$ CD43$^-$ CD1c$^-$), and B-1 (CD20$^+$ CD27$^+$ CD43$^+$ CD1c$^-$) B cells for 5 days in the presence of immobilized anti-CD3 antibody. Concentrations of human IgM and IgG in the culture supernatant were compared among the B cell alone group (light gray), the CD49d^{low} CD4$^+$ T cell group (white), the CD49d^{high} CD4$^+$ T cell group (black), and the CXCR5$^+$ CD4$^+$ follicular help T cells (dark gray). Data are expressed as the mean ± standard error of the mean (n = 3 donors per group). *$P < 0.05$, **$P < 0.01$; Student's *t*-test. SP, spleen; PB, peripheral blood.
